# Persistent Expectoration of Pulmonary Artery Embolization Coils Requiring Lobectomy

**DOI:** 10.1016/j.atssr.2023.11.017

**Published:** 2023-12-09

**Authors:** Laura DiChiacchio, Gregory A. Grandio, David C. Griffin

**Affiliations:** 1 Division of Cardiothoracic Surgery, University of Utah, Salt Lake City, Utah; 2 Division of Pulmonary Critical Care Medicine, Intermountain Medical Center, Salt Lake City, Utah; 3 Division of Cardiothoracic Surgery, Intermountain Medical Center, Salt Lake City, Utah

## Abstract

Transcatheter embolization is an important tool in the treatment of pulmonary artery and bronchial artery pseudoaneurysms, malformations, and hemorrhage. Migration of coils, particularly through erosion into the associated bronchus, is a rare but known potential complication after the use of intravascular embolization coils in the pulmonary vasculature. Here we present a case of migration of segmental pulmonary artery coils through the right lower lobe bronchus and proximally through the vocal cords, causing significant symptoms of discomfort, hemoptysis, and ongoing expectoration of coils necessitating thoracoscopic right lower lobectomy.

Coil embolization is used in the treatment of multiple pulmonary vascular anomalies including pulmonary artery (PA) pseudoaneurysms. Coil migration into the nearby bronchus is a rare but described complication, usually manifested in a delayed fashion. Attempts at wire retrieval can be performed when coil migration into the airway occurs, but this can be unsuccessful and lead to surgical removal through lobectomy or segmentectomy.

A COVID-19–associated necrotizing pneumonia developed in a man in his late 50s in 2021, with symptoms progressing to massive hemoptysis. Computed tomography pulmonary angiography demonstrated a right lower lobe segmental PA pseudoaneurysm. He underwent coiling of the pseudoaneurysm by interventional radiology with resolution of his symptoms. One year after coiling, he presented with low-volume hemoptysis, expectoration of a short, thin piece of wire, and a vibrating sensation in his airway. He underwent bronchoscopy, which demonstrated erosion of the PA embolization wires into the airway with migration through the vocal cords (Figure 1). A portion of the wire was removed, but it could not be extracted in its entirety. Despite multiple attempts to extract the eroded wires bronchoscopically, complete removal was not possible, primarily because of fracturing of the wires during attempted retrieval.

**Figure 1:**
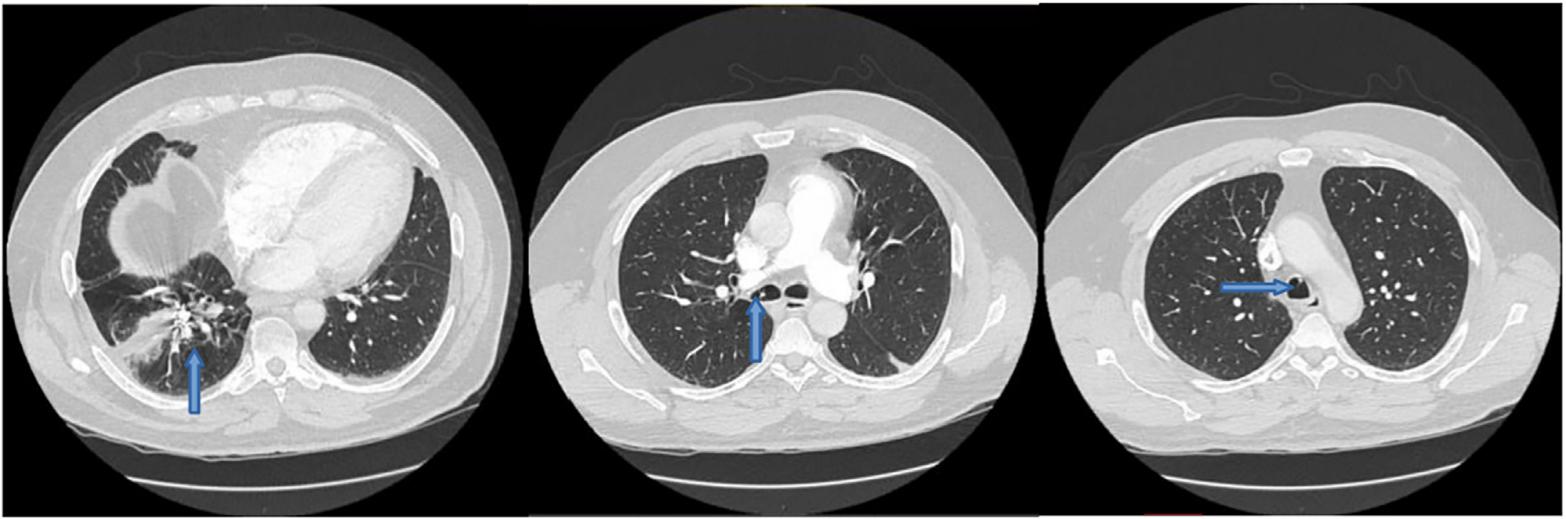
Computed tomography pulmonary angiogram with embolization coils in right lower lobe segmental pulmonary artery with extension into the trachea (arrows).

The patient remained symptomatic, in constant discomfort with continued low-volume hemoptysis. He was offered a video-assisted thoracoscopic right lower lobectomy with complete removal of all coils. Preoperative computed tomography angiography demonstrated the expected location of the embolization coils (Figure 2). Minimally invasive right lower lobectomy was undertaken, and he had an uneventful recovery with complete resolution of his symptoms. On intraoperative bronchoscopy, the only notable finding was visible wire coming from the right basilar segment and extending to the vocal cords. Bronchoscopic confirmation that there was no residual wire present after lobectomy was performed. Gross pathologic examination revealed a right lower lobe specimen with pleural adhesions and multiple individual submillimeter wires, with several extending into the right lower lobe bronchus. The tissue surrounding the bundle of wires was found to be consolidated and atelectatic with an adjacent 1-cm abscess cavity. Some of the wires from the bundle were found to enter the abscess cavity and were resected en bloc. He was able to be discharged on postoperative day 3 with no significant complications, and on postoperative clinic visit, his symptoms had resolved.

**Figure 2:**
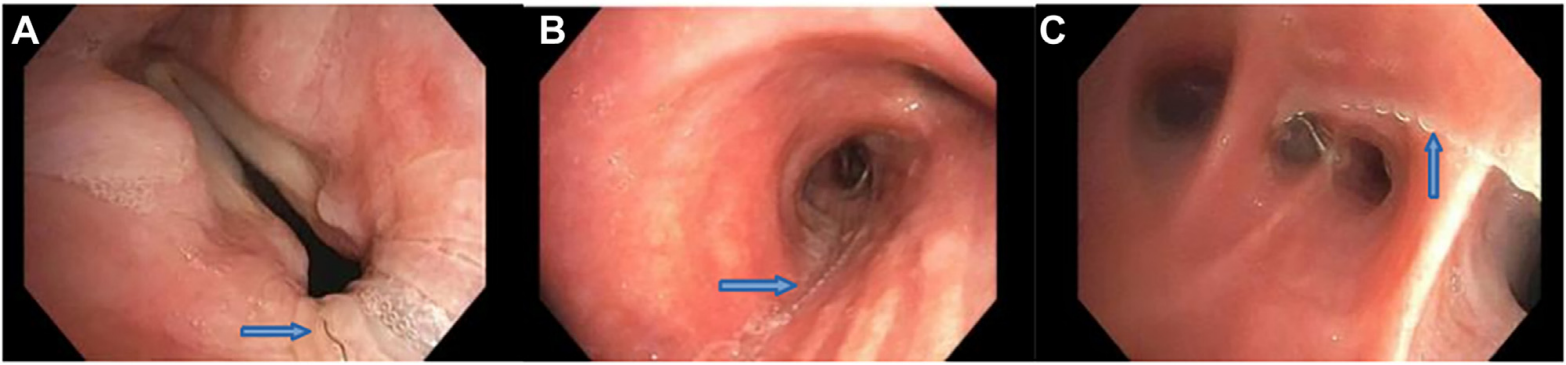
Bronchoscopic images. (A) Thin, dark portion of wire protruding through vocal cords (arrow). (B) Thicker portion of wire in right mainstem bronchus (arrow). (C) Thicker portion of wire followed down to right lower lobe airway (arrow).

## Comment

Coil embolization of multiple pulmonary vascular pathologic conditions is performed with regularity, with overall excellent outcomes. PA vascular malformation embolization, for example, has a >99% success rate with a reported incidence of embolization-related complications of <0.7%.^1^^,^^2^ In an early series of hemoptysis secondary to coil migration after PA vascular malformation embolization, 3 reported cases progressed to requiring surgical lobectomy.^2^, ^3^, ^4^, ^5^ In a report of traumatic PA pseudoaneurysm embolization with associated review of the literature, most cases of PA pseudoaneurysms require surgical resection through either lobectomy or segmentectomy, although reports of rigid or flexible bronchoscopic removal do exist.^6^, ^7^, ^8^ Consensus exists that refractory hemoptysis secondary to coil migration requires full extraction.^6^ Whether this is performed by bronchoscopic retrieval or surgical resection depends on patient-specific factors. In the case presented here, bronchoscopic retrieval was attempted but not possible, given the location and fragility of the embolization coils. Therefore, minimally invasive lobectomy was pursued, and the patient has had an excellent short-term outcome.
